# Interpretation of the HLA epitopes—Continuous dilution method for detection of high‐titer IgG HLA antibody

**DOI:** 10.1002/jcla.23632

**Published:** 2020-12-01

**Authors:** Yanli Xiao, Wei Liu, Zhongyu Kang, Daihong Li

**Affiliations:** ^1^ Department of Blood Transfusion Tianjin First Central Hospital Tianjin China

**Keywords:** donor‐specific HLA antibodies, epitope, titration study

## Abstract

**Background:**

The presence or absence of human leukocyte antigen (HLA) antibodies, especially the strength of donor‐specific HLA antibodies (DSAs), has important roles in clinical evaluation and diagnostic decision‐making for solid‐organ transplantation. Dilution patterns help to give a new sight of HLA epitopes. “Epitope matching” is likely to lower the risk of developing DSA and increase the likelihood of matching a compatible donor.

**Methods:**

We collected data evaluating HLA antibodies with a titration study using mean fluorescence intensity.

**Results:**

Diluting the serum of recipients can reduce potential inhibitory effects, accurately evaluate the intensity of donor‐specific HLA antibodies, and guide surgeons to take or not take intervention measures. Dilution patterns also help to give a new sight of HLA epitopes.

**Conclusion:**

We believe that from the viewpoint of HLA antibodies, the dilution model can provide new tools and insights for the study of HLA epitopes.

## 1 INTRODUCTION

Donor‐specific human leukocyte antigen (HLA) antibodies before or after transplantation may have different effects based on antibody strength. This feature may increase the risk of antibody‐mediated rejection and lose valuable opportunities for transplantation, affect graft survival, and even lead to graft failure. A history of sensitization before renal transplantation, including a history of blood transfusion, pregnancy, secondary transplantation, and other risk factors, may lead to the production of donor‐specific HLA antibodies (DSAs) in the body.^1^ Why some recipients develop DSAs to their HLA‐mismatched donor and others do not is not known. Recently, “epitope matching” has entered the lexicon, but what contributes to antigenic epitopes is incompletely understood.

In recent years, computer software called “HLAMatchmaker” (www.epitopes.net/) has become increasingly popular. HLAMatchmaker identifies the number of differences in amino acid sequences between the donor and recipient HLA antigens, which are called “eplets”. HLAMatchmaker has been used in several studies to calculate “eplet loads” (ie, the overall difference in HLA class‐II amino acid sequences between donors and recipients). Tambur and colleagues clearly showed that determination of the eplet load was a powerful tool for risk stratification of patients to understand their likelihood of producing HLA antibodies and to individualize immunosuppression and post‐transplant monitoring.^2^ In many cases, especially if the recipient is highly sensitized or the antibody pattern is complex, HLAMatchmaker cannot give a clear epitope load.

The HLA single‐antigen microbead (SAB) method based on the Luminex platform is the most sensitive method for the detection and semi‐quantitative identification of anti‐HLA antibodies and evaluation of immune risk of organ transplantation.^3^ Particularly for DSA detection before or after transplantation, SABs may have a different mean fluorescence intensity (MFI) because of the prozone phenomenon, and DSA interpretation may have different meanings according to the MFI of the antibody, which may affect clinical decision‐making. The specificity of antibodies in highly sensitized recipients is, in general, difficult to explain, but it may help to reveal some characteristics of HLA epitopes.

This report is aimed at studying HLA epitopes. The specific antibody epitopes can be better identified by the true strength of the antibodies obtained by continuous dilution. The use of “HLAMatchmaker” (www.epitopes.net/) epitope registration system to screen these mismatched epitopes and recipient HLA alleles corresponding to mismatched epitopes is helpful for clinical selection of suitable donors.

## 2 CASE REPORT

A 45‐year‐old man was diagnosed with chronic renal failure and underwent allogeneic kidney transplantation in March 2012. The patient took tacrolimus, mycophenolate mofetil, and hormone immunosuppressants regularly after transplantation. During follow‐up, his renal‐function index showed creatinine at 1200 Umol/L. Renal biopsy revealed chronic active rejection which is mediated by T cells in renal allografts. HLA antibodies showed negative anti‐HLA class‐Ⅰ and strong positive anti‐HLA class ‐Ⅱ. The clinical diagnosis was graft failure. After desensitization treatment on March 2019, HLA class‐Ⅰ and ‐Ⅱ antibodies were detected routinely using the SAB method.

Human leukocyte antigen typing was undertaken using a LABType^®^ kit (One Lambda). Testing of HLA antibody was undertaken using a LifeCodes^®^ Single Antigen kit (Immucor). Both methodologies followed manufacturer guidelines, as described previously.^4^

**FIGURE 1:**
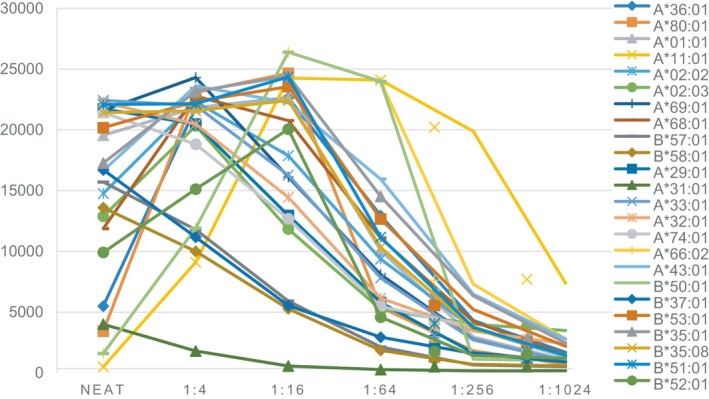
Comparison of the strength of HLA‐Ⅰ antibodies with different dilutions. The *X* axis represents different dilutions, and the *Y* axis represents the MFI. The HLA typing in our patient was: HLA‐A24, A30; B13, B60(40); DR7, DR11; DQ2, DQ7. The donor HLA typing was: HLA‐A11, A24; B13, B35; DR12, DR15; DQ6, DQ7. The DSAs were: A*11:01, B*35:01. They have strong inhibitory effects in neat serum

The MFI of most of the SABs of our patient was >15 000, and the pattern of the cross‐reaction group was disordered (Figure 1). Testing of the neat antibody indicated strong interference effects, with MFI values not correlating with the true strength of the antibody. In the specific reaction of an antigen and antibody, only when the proportion of antigen and antibody is appropriate can an antigen‐antibody complex be formed. If the amount of antibody is much larger than that of antigen, the binding of antigen and antibody is not at an optimal proportion. The continuous dilution method was used to describe a peak MFI > 5000 in which 24 HLA‐A and ‐B‐coated SABs were positive. Different antibodies have different dilution patterns, which may be of higher or lower strength than the initial test. These factors led to the prozone phenomenon in serum to have distinct effects on different antibodies.

**FIGURE 2:**
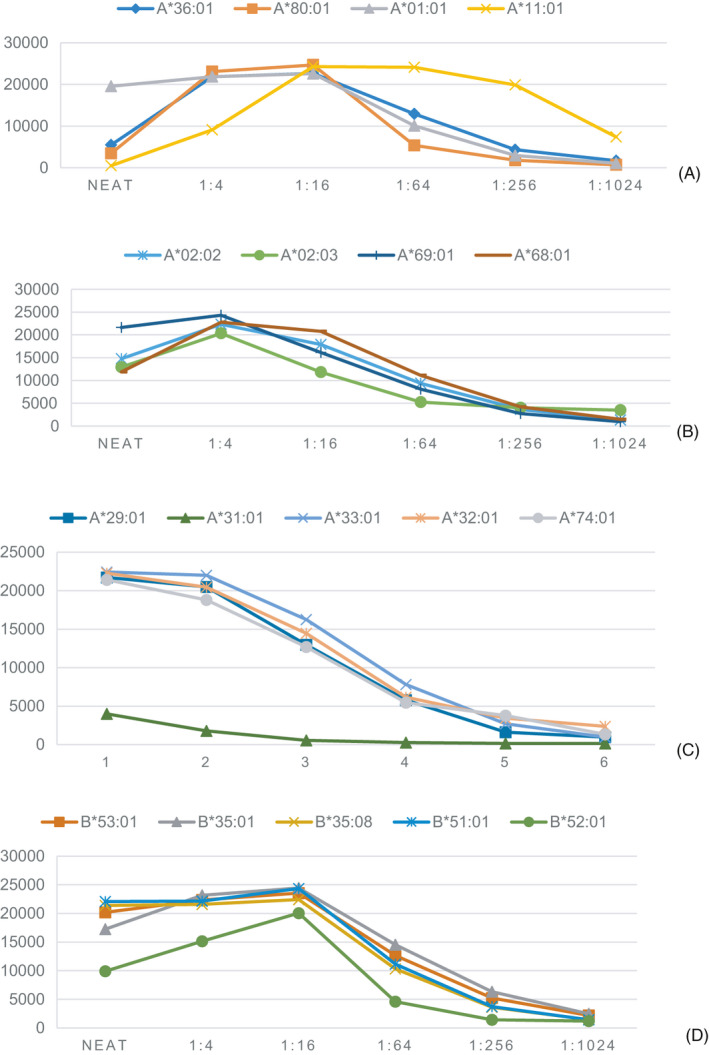
Four cross‐reactivities separated from Figure 1. The cross‐reaction group at the HLA‐A11 (A) HLA‐A2 cross‐reactive group (B) HLA‐A19 cross‐reactive group (C) HLA‐B35 cross‐reactive group (D)

We divided all loci into several specific clusters, which are revealed separately in Figure 2A‐D. The latter shows quite unique dilution patterns for each cross‐reactive group. Figure 2A illustrates four HLA‐Ⅰ alleles showing strong inhibition in neat serum, with a top MFI of ~22 000 at a titer 1:4‐1:16. When the titer reached 1:1024, the antibodies were negative, except for A*11:01. Figure 2B shows the patient developing antibodies to a HLA‐A2 cross‐reactive group. The four quite similar dilution curves reached a peak MFI of titer 1:4. During titration, the antibody strength weakened gradually. There were several types of decomposition products of A*19, including A*29, A*31, and A*32. All antibodies in Figure 2C illustrated seemingly similar inhibitory effects, and all had a peak MFI of ~23 000. Figure 2D is a compilation of the B35 cross‐reactivity antibodies and shows quite similar dilution curves. MFI analyses of the sites of the cross‐reaction groups mentioned above revealed that the dilution patterns of the sites located in the same cross‐reaction group were approximately identical.

The interpretation of epitope names consists of polymorphic residues with different sequence location numbers and standard single‐letter amino acid codes. For example, 150 V means that the sequence position of 150 is valine. Some antigens have at least two unique epitopes at different sequence positions. For example, A1 and A36 alleles share three unique epitopes: 44 KM (described by 44Q, 44K, 45m, and 46e), 149A + 150V + 151H and 158V. The epitope symbol is 44KM3, which indicates the possibility of three different epitopes. These epitopes can be predicted as recognition sites of specific antibodies and have been verified by experiments with informative specific antibodies. Each HLA antigen has its own unique combination of epitopes, but at the same time, many of these epitopes are shared with other HLA antigens. Through interpretation of the cross‐reaction group and antibody‐dilution model, the antigenic epitopes described in Table 1 located in the same cross‐reaction group were found to be quite similar.

**Table 1:** The epitope specificity of HLA antibodies

	Neat	1:4	1:16	1:64	1:256	1:1024
A*36:01	9F(ABC)	65RA	65RA	65RA	65RA	151H
A*80:01	207S,9F(ABC)	65RA	65RA	65RA	65RA	/
A*01:01	9F(ABC)	65RA	65RA	65RA	65RA	151H
A*11:01	/	65RA	65RA	65RA	65RA	151H
A*02:02	207S,9F(ABC)	65RA	65RA	65RA	65RA	151H
A*02:03	207S,9F(ABC)	65RA	65RA	65RA	65RA	151H
A*69:01	207S,62RN + 163TW	207S,62RN + 163TW	207S,62RN + 163TW	207S,62RN + 163TW	65RA	151H
A*68:01	207S,62RN + 163TW	207S,62RN + 163TW	207S,62RN + 163TW	207S,62RN + 163TW	65RA	151H
B*53:01	131S	131S	131S	131S	163LW + 65QI,66IF	163LW + 65QI
B*35:01	131S	131S	131S	131S	163LW + 65QI,66IF	163LW + 65QI
B*35:08	131S	131S	131S	131S	163LW + 65QI,66IF	163LW + 65QI
B*51:01	131S	131S	131S	131S	163LW + 65QI,66IF	163LW + 65QI
B*52:01	131S	131S	131S	131S	163LW + 65QI	163LW + 65QI

**FIGURE 3:**
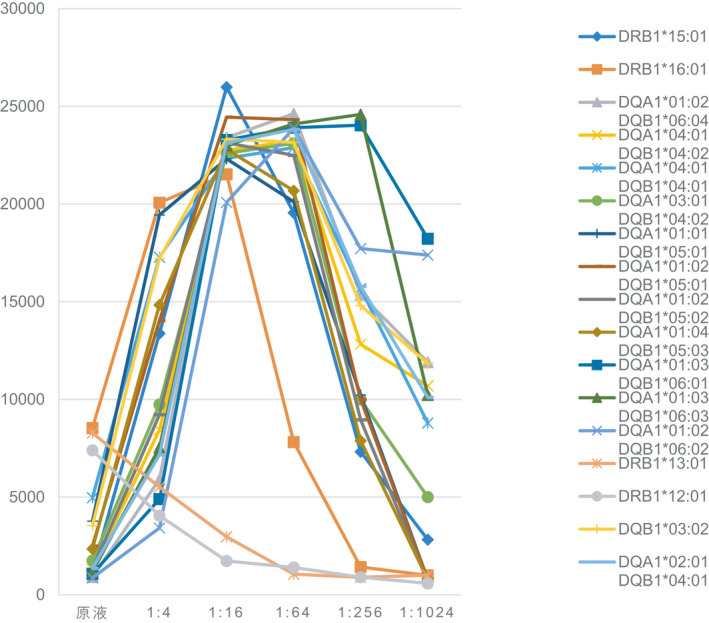
Comparison of the strength of HLA‐Ⅱ antibodies at different dilutions. The X axis represents different dilutions. The Y axis represents the MFI

**FIGURE 4:**
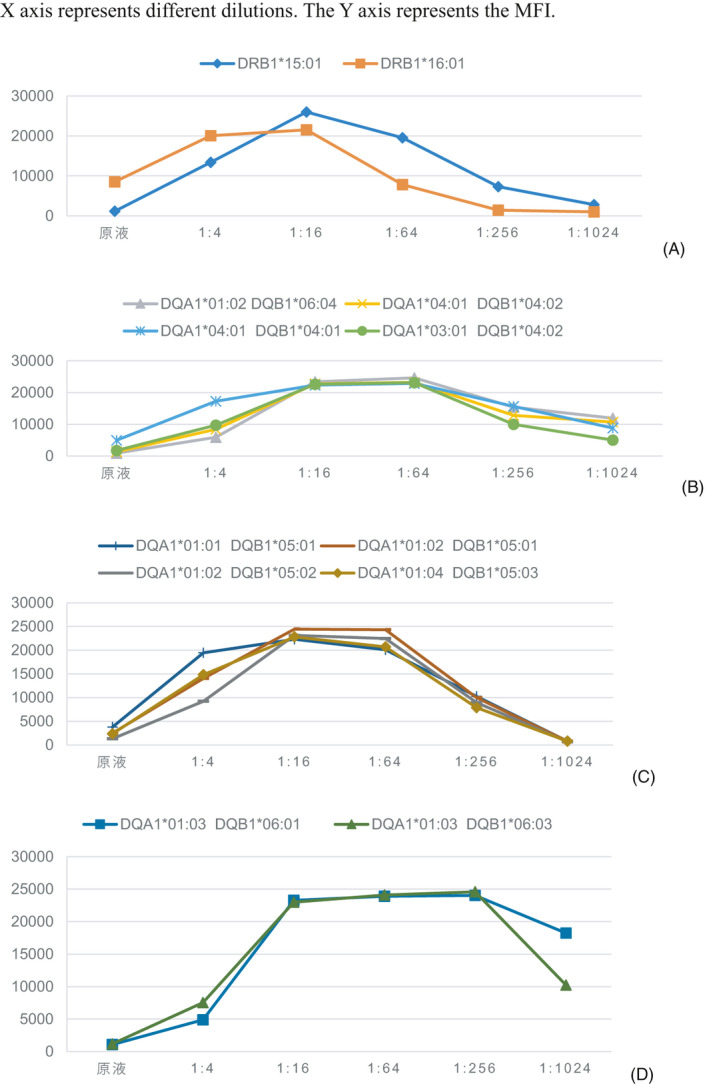
Four cross‐reactivities separated from Figure 3. The broad‐specific cross‐reaction group of HLA‐DR51(A) HLA‐DQ alleles(B‐D)

Figure 3 shows the specificity of class‐II antibodies in our patient. A titration study of HLA class‐II antibody revealed the same characteristics as that for class Ⅰ. That is, the specificity of some sites had the highest MFI at one titer, whereas the specificity of other sites had the highest MFI at another titer. Specifically, in the broad‐specific cross‐reaction group of HLA‐DR51 in Figure 4A, the titration pattern of DRB1*15:01 and DRB1*16:01 was similar, and some inhibitory factors in serum had the same inhibitory effect on the two antibodies, showing the highest MFI at 1:16 dilution.

Figure 4B,C shows another group of HLA‐DQ alleles that seem to be expected: DQA1*01:02/DQB1*06:04, DQA1*04:01/DQB1*04:02, DQA1*04:01/DQB1*04:01, DQA1*03:01/DQB1*04:02,DQA1*01:01/DQB1*05:01, DQA1*01:02/DQB1*05:01, DQA1*01:02/DQB1*05:02, and DQA1*01:04/DQB1*05:03. This group of alleles began with a low MFI and reached a peak MFI at 1:64 titer (MFI was 17000/20000).

An almost identical dilution pattern indicated that these sites had a common epitope.

Figure 4D shows that two HLA‐DQ alleles had strong inhibition in undiluted samples. The peak MFI of DQA1*01:03/DQB1*06:01 and DQA1*01:03/DQB1*06:03 was ~13 000, both. When the antibody was diluted to a certain concentration, the antibody titer out (turned negative). Some features seemed to follow the current convention of naming HLA‐DQ antibodies. Our data suggested that DQA1*01:03 encoded a common target/epitope.

## 3 DISCUSSION

In recent years, explaining what contributes to an HLA B‐cell epitope has proved to be limited and inadequate, as has understanding of the immunogenicity and antigenicity of mismatched HLA epitopes. The concept of epitopes on different HLA antigens has been extended to structural eplets. These epitopes themselves are determined by a small number of adjacent amino acids.^5^ The high level of sharing of antigenic epitopes among HLA molecules is the reason why HLA antibodies produced by individuals exposed to a single HLA antigen can also react with other, unexposed HLA antigens. Compared with a single different antigen, it is more accurate to regard any given HLA molecule as a common and unique set of antigenic epitopes.^6^

We provided data suggesting that MFI values for neat antibody do not always depict antibody strength accurately. A dilution study provides more dynamic monitoring of antibody behavior, and it is a more suitable method for measurement of antibody strength. A dilution method can better describe the antigen‐antibody reaction. Titration studies, on the one hand, show information about current antibody‐naming groups and, on the other hand, provide a tool to decipher antibody reactivity. According to the definition of a dilution pattern, antibodies can provide clues about shared antibody targets, thereby providing clues to physiologic epitopes. Application of an antibody titer for detection of HLA antibodies undoubtedly increases the economic burden of patients. According to the experience of our center, we can choose the original serum plus two additional dilutions (eg, 1:16 and 1:64) to obtain the trend of the antibody reaction. Dilution methods are recommended to detect HLA antibodies in highly sensitized patients undergoing a secondary transplant.

Kosmoliaptsis and colleagues ^7^ pointed out that the exact mechanism of the interference effects observed in detection of monoclonal antigens using the Luminex platform is not completely clear, and that it may be multifactorial. Compared with serum‐pretreatment methods such as ethylenediamine tetraacetic acid and C1q status,^8^ the dilution method can better solve the prozone phenomenon.

Epitope matching minimizes the risk of developing de novo HLA‐DSAs after transplantation. Therefore, solving the mystery of HLA B‐cell epitopes, understanding HLA epitopes, and deciphering the factors that determine the antigenicity and immunogenicity of epitopes may elicit substantial changes to solid‐organ transplantation. We believe that from the viewpoint of HLA antibodies, the dilution model can provide new tools and insights for the study of HLA epitopes.

## ETHICAL APPROVAL

Written informed consent was obtained from the patient to use his data. Approval from the ethics board of Tianjin First Central Hospital was not required.
